# Evaluation of pulpal response at varying remaining dentin thickness in teeth restored with resin bulk fill composite, conventional glass ionomer cement and silver amalgam: Histomorphometric analysis

**DOI:** 10.1016/j.jobcr.2024.12.020

**Published:** 2025-02-12

**Authors:** Ankita Agarwal, Promila Verma, Rakesh Yadav, Ramesh Bharti, Rhythm Bains, Madhu Kumar, Dipti Shastri

**Affiliations:** a Department of Conservative Dentistry and Endodontics, Faculty of Dental Sciences, King George's Medical University, Lucknow, India; b Department of Pathology, King George's Medical University, Lucknow, India; c Department of Orthodontics & Dentofacial Orthopaedics, Faculty of Dental Sciences, King George's Medical University, Lucknow, India

**Keywords:** RDT, SDR BULK-FILL FLOWABLE COMPOSITE, GIC, AMALGAM, HISTOMORPHOMETRIC ANALYSIS, INFLAMMATORY RESPONSE

## Abstract

**Objective:**

To compare and evaluate the pulp response to GC glass ionomer cement, SDR plus bulk fill composite and amalgam against gold standard calcium hydroxide cement at varying remaining dentin thickness, in teeth planned for orthodontic extraction.

**Method:**

Thirty-eight human premolars were prepared with 2 mm or 2.5 mm depth cavities. They were restored with GC conventional glass ionomer cement, SDR plus bulk fill composite, amalgam, or lined with Dycal and restored with GIC. Two teeth were used as intact controls. After a 7-day interval, the teeth were extracted and processed for histological examination of the pulp and the thickness of the remaining dentin between the cavity floor and pulp tissue.

**Results:**

All experimental groups showed some degree of inflammatory response. A significantly higher inflammatory response and more tissue disorganization were observed with SDR bulk fill composite (p < 0.05) compared to Glass ionomer cement, amalgam and Dycal at both cavity depths of 2 mm or 2.5 mm. The mean RDTs ranged from 346 μm to 1025 μm.

**Conclusions:**

The study concluded that critical RDT varies for different restorative materials. It was observed that both glass ionomer cement and amalgam demonstrated acceptable biocompatibility when used in deep cavities. At the same time, SDR plus bulk fill composite proved to be the least biocompatible.

## Highlights

• Inflammatory cell response at 2 mm cavity depth **(RDT:1mm-0.5 mm)**. SDR plus bulk fill composite > GIC = Amalgam > Control.
• Inflammatory cell response at 2.5 mm cavity depth **(RDT: <0.5 mm)**. SDR plus bulk fill composite > Amalgam > GIC > Control.
• Tissue Disorganization at 2 mm cavity depth **(RDT: 1mm-0.5 mm)**. SDR plus bulk fill composite > GIC > Amalgam > Control.
• Tissue Disorganization at 2 mm cavity depth **(RDT: <0.5 mm)**. SDR plus bulk fill composite > Amalgam = GIC > Control.

## Graphical abstract

**Figure:**
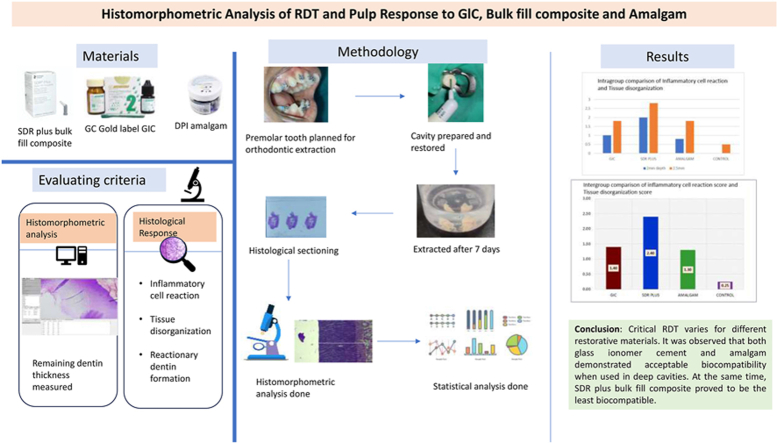

## 1 Introduction

Dental caries and trauma often necessitate restorative procedures to restore tooth function and aesthetics. The choice of restorative material and the remaining dentin thickness (RDT) after tooth preparation influences the success of these restorations. RDT, the healthy dentin between the cavity's pulpal floor and the pulp, is crucial for pulpal health and long-term restoration success.^1^^,^^2^ Reduced RDT can lead to pulpal inflammation due to increased permeability, allowing bacteria and byproducts from restoratives to reach the pulp.^3^

Previous studies have underscored the importance of preserving dentin thickness to prevent pulpal irritation and inflammation.^2^^,^^4^^,^^5^ However, there still needs to be more consensus regarding the optimal RDT for different restorative materials, particularly in the context of newer materials such as SDR bulk fill composite. This study's findings could fill this gap, offering a more comprehensive understanding and paving the way for improved restorative procedures.

Minimizing pulp injury during cavity preparation and restoration is essential for maintaining pulp cell function and viability and reducing postoperative complications. To achieve this, assessing the potentially harmful effects of cavity preparation, conditioning, and restoration on pulpal viability and function is essential. Previous studies have evaluated pulp response by examining several histological outcomes.^6^, ^7^, ^8^, ^9^, ^10^, ^11^. Deep cavities thin the dentin layer, creating pathways, such as large open dentinal tubules, through which cytotoxic substances like HEMA and TEGDMA from resin cement, phosphate ions from zinc polycarboxylates and zinc phosphates can penetrate and reach the pulp tissue causing pulpitis.

Commonly used restorative materials such as glass ionomer cement (GIC), amalgam, and bulk fill composites like SDR each have unique properties that can influence pulpal response. For example, GIC can induce a mild dentinogenic response without significant harm to odontoblasts.^8^ While concerns about the mercury content of amalgam have been raised, some research suggests that dental amalgam does not pose significant health risks to the general population. This assertion is backed by systematic reviews from several international health organizations, including the U.S. FDA.^12^^,^^13^. These reviews have concluded that there is not enough evidence to connect mercury exposure from dental amalgam to harmful systemic health effects.

This study is significant as it aims to evaluate the histological response of dental pulp to varying RDTs in teeth restored with GIC, amalgam, SDR plus bulk fill composite, and Dycal. In relation to bulk fill composite's properties and biocompatibility, many in vitro studies are present, but there is a lack of clinical studies. This ex vivo study provides data to fill that gap. It also provides crucial insights into the optimal RDT for these materials, thereby enhancing clinical decision-making and, ultimately, the longevity of dental restorations.

## 2 Material and methods

### 2.1 Participant selection

During this study, 40 premolars designated for extraction from orthodontic patients at the Department of Orthodontics and Dentofacial Orthopaedics, were selected for analysis. The average age of the participants was 17 years. Detailed explanations and reading materials about the experiment, encompassing its objectives, procedures, and potential hazards, were provided to the participants and their parents/guardians. Ethical clearance was obtained from the Institutional Human Subjects Ethics Committee (Ref. code: XVII-PGTSC-IIA/P5), and the parents/guardians and the participants duly signed the consent forms before the procedure, after being informed. During the examination of the diagnostic and pre-extraction radiographs, certain teeth were excluded from the study based on specific criteria. This included the presence of proximal or root caries, existing restorations, immature apices, internal or external resorption, and other pathological conditions that could influence the assessment of pulpal response.

### 2.2 Group allocation

**Fig. 1:**
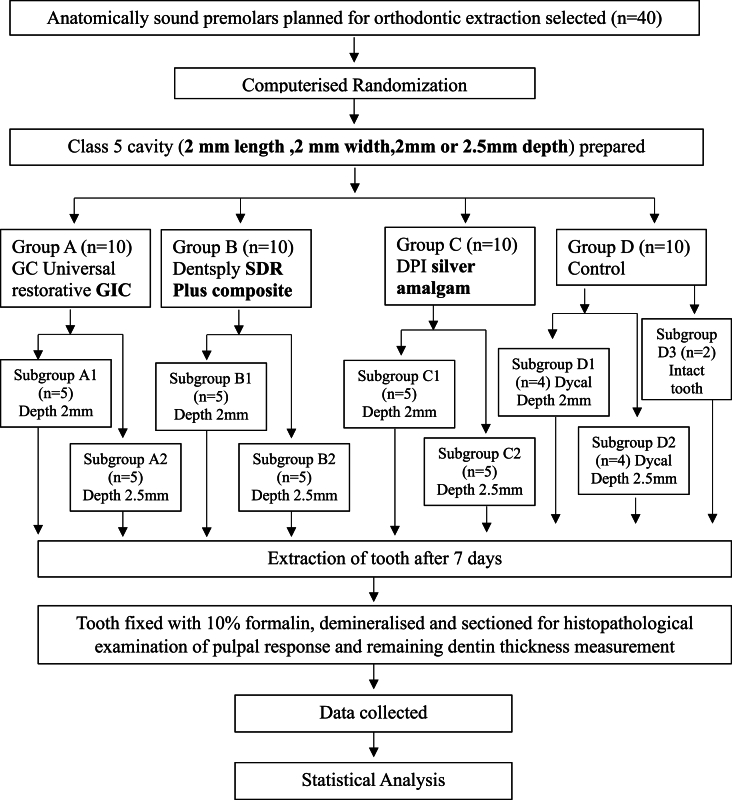
Illustration of study design.

The teeth were divided into four groups according to the material used to restore them and further into subgroups according to the cavity depth (Fig. 1). To achieve a split-mouth design in line with the study's objective, randomization software (random allocation software 2.0) was used. Two teeth per group were filled with the same material at different depths, so block randomization with an equal block size of 20 samples was done.

### 2.3 Cavity preparation

**Fig. 2:**
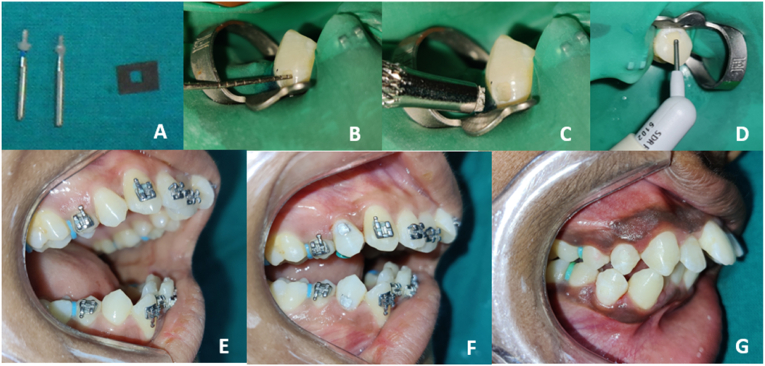
Clinical procedure of cavity preparation and restoration, A) Burs with resin stop, cavity template, B) Prepared cavity, C) and F) cavity restored with amalgam, D) and G) cavity restored with SDR plus bulk fill composite, E) Pre operative photograph of patient.

A single operator performed all the restorative procedures to prevent interoperator bias. Prior to administering local anaesthesia, the operator rinsed the oral cavity with a 0.12 % chlorhexidine solution.^6^, ^7^, ^8^^,^^14^ The tooth was cleaned using a pumice slurry applied with the help of a rubber cup, followed by preparation of Class V cavities on the buccal surface of each tooth with a high-speed handpiece and ample water cooling.^15^^,^^16^ A resin stop was affixed to straight fissure diamond points to ensure a 2.5 mm or 2.0 mm standard cavity depth(Fig. 2a), which the operator replaced after every four-cavity preparation. The final cavity dimensions were 2.0 mm in length, 2.0 mm in width, and 2.5 mm or 2 mm in depth with no undercuts (Fig. 2b).

### 2.4 Cavity restoration

In **group A**, the cavity floor underwent a 10-s treatment with a 25–30 wt% polyacrylic acid solution sourced from GC, Tokyo, Japan. Subsequently, the enamel was air-dried, and the dentin was meticulously dried with sterile absorbent paper. The operator filled the cavity with self-cure Glass Ionomer Cement (Gold Label 2, Universal Restorative GIC, GC, Tokyo, Japan) to a depth of either 2 mm (Subgroup A1) or 2.5 mm (Subgroup A2). The setting time for the cement was 8 min.

In **group B**, all cavities were etched using 37 % phosphoric acid (Eco-etch, Ivoclar, Zurich, Switzerland) for 15 s to enhance marginal integrity, as pulp reactions to acid etchants are generally mild to moderate.^11^^,^^17^ After thoroughly rinsing and drying the cavity, a bonding agent (Vivadent Te-Econom Bonding Agent, Ivoclar, Zurich, Switzerland) was applied, followed by curing for 20 s using an LED curing light (LED D, Woodpecker, China). A radiometer confirmed that the device's light intensity was not less than 400 mW/cm2, as the low intensity can lead to poor curing capacity.^18^ The operator restored the teeth with SDR Plus bulk fill composite (Dentsply Sirona, USA) and cured them for 40 s at a depth of 2 mm (Subgroup B1) or 2.5 mm (Subgroup B2) (Fig. 2d and g).

In **group C**, amalgam (DPI, Mumbai, India) manipulations were performed using an automatic amalgamator for each mix (0.06 g of alloy triturated with 0.06 g of mercury for 10 s). The cavities were incrementally filled to the depth of 2 mm (Subgroup C1) or 2.5 mm (Subgroup C2), followed by incremental hand condensation, carving and finishing of the restoration (Fig. 2c and f). Since the same operator conducted all procedures, the condensation pressure was assumed to be consistent across all cavities, ensuring a uniform pulp response. All amalgam restorations were intentionally left unpolished to avoid unintentional changes in the pulp response due to polishing. During the restoration process, the aim was to complete as much finishing work as possible while carving.^15^

In **subgroup D1 and subgroup D2**: The cavity floor at depths of 2 mm and 2.5 mm, respectively, were lined with hard-setting calcium hydroxide cement (Dycal, DENTSPLY Caulk Milford, DE, USA). Adhesive cavity restorations with GIC were then performed as described above. Two healthy teeth without any cavities were included as intact controls (**subgroup D3**) to verify the reliability and accuracy of the laboratory procedures for all teeth used in this study (negative control).

### 2.5 Tooth extraction

Seven days after the clinical procedures, the teeth were extracted atraumatically under local anaesthesia in the Oral and Maxillofacial Surgery Department. The oral surgeon positioned the extraction forceps close to the tip of the root to avoid causing changes to the inner pulp. The teeth were immediately stored in 10 % formalin solution and transported to the Department of Conservative Dentistry and Endodontics. The roots were sectioned transversely 6 mm from the CEJ using a high-speed handpiece with a tapered fissure carbide bur (H267, Prima Dent) under water spray to enhance fixative penetration.

### 2.6 Histopathology

**Fig. 3:**
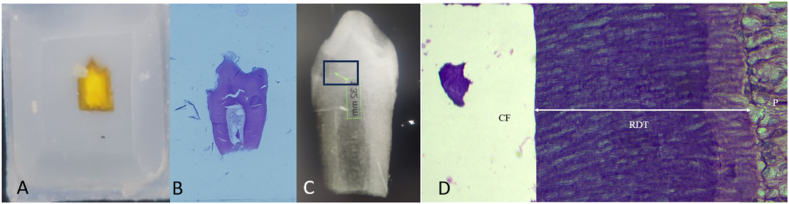
Histopathological examination. A) Restored tooth embedded in resin block, B) H/E stained slide of tooth section, C) Radiograph of restored extracted tooth displaying RDT, D) Histomorphometric analysis of histological tooth section where cavity floor (CF), remaining dentine thickness (RDT) and pulp (P) are marked.

The teeth were immersed in a formalin fixative solution for 48 h at pH 7.2, then underwent decalcification in a 5 % nitric acid solution, dehydrated, vacuum infiltrated with wax paraffin, and were embedded in paraffin (Fig. 3a). Subsequently, serial sections with a thickness of 4 μm were produced, mounted on glass slides, and stained with hematoxylin and eosin (H/E)(Fig. 3b). Consistent with previous procedures,^6^, ^7^, ^8^, ^9^, ^10^, ^11^ a single examiner, blinded to the samples, assessed all sections using a light microscope (Nikon Eclipse E200, Tokyo, Japan). The examiner recorded the histopathological characteristics according to the criteria delineated in a previous study by *Ribeiro* et al.^8^

The remaining dentin thickness (RDT) between the cavity floor and the pulp chamber was quantified for each tooth utilizing a Nikon Eclipse light microscope connected to a Samsung Digital Camera SSC/131. Subsequently, the captured images were transferred to a computer for analysis using Mosaic V2.0 software (Fig. 3c).

### 2.7 Statistical methods

Analysis of Variance (ANOVA), which accounts for comparing three or more independent groups, was used to find statistical differences in the means of remaining dentin thickness in intergroup comparisons.

Since the operator dealt with a discrete outcome variable (score), four independent groups, and a small sample size which didn't assume a normal distribution, the Kruskal-Wallis test was used to assess differences in inflammatory cell reactions, tissue organization and reactionary dentin formation categories. The Dunn-Bonferroni post-hoc test compared multiple pairs in a data category.

Wilcoxon signed-rank test, which compares two sets of scores from the same participants, was used for intragroup comparison of the mean histological outcomes at the two cavity depths (2 mm and 2.5 mm). The statistician used the software IBM SPSS Statistics 23 (IBM Corp., Armonk, N.Y., USA) for all analysis.

## 3 Results

The participants did not report any specific symptoms or pain during the study. Prior to the clinical procedures, radiographic assessments of all teeth involved revealed no signs of periapical pathology. Additionally, radiographs taken immediately before extraction at the 7-day interval did not show any changes in the periapical area of the restored teeth. Table 1 provides a histomorphometric measurement of each cavity's remaining dentin thickness (RDT). Table 2 presents the number of teeth in the groups with a specific histological outcome score at both cavity depths.

**Table 1:** Histomorphometric analysis of Remaining dentin thickness (RDT, μm).

Group	Specimen	Cavity Depth	
		2000 μm (2 mm)	2500 μm (2.5 mm)
Glass ionomer cement (A)	1	1023	505
	2	1200	688
	3	543	132
	4	801	344
	5	910	266
Mean ± standard deviation		895.40 ± 246.16^a^	387.00 ± 215.66
SDR plus bulk fill flowable composite (B)	1	924	413
	2	901	288
	3	1046	512
	4	895	301
	5	835	302
Mean ± standard deviation		920 ± 77.63	363.20 ± 97.33
Silver Amalgam (C)	1	1044	564
	2	1023	301
	3	937	427
	4	801	215
	5	695	225
Mean ± standard deviation		900 ± 149.21	346.40 ± 148.25
Dycal (D)	1	1002	289
	2	1195	621
	3	805	312
	4	1100	512
Mean ± standard deviation		1025.50 ± 166.79	433.50 ± 160.17

a RDTs are not statistically different among groups at specified same cavity depths (ANOVA, p > 0.05).

**Table 2:** Number of teeth for each score according to groups and cavity depth.

Histopathological event	Group		Cavity				Depth				Total Number of teeth
			2 mm				2.5 mm				
		score	0	1	2	3	0	1	2	3	
Inflammatory reaction (ICR)	Glass ionomer cement (A)		2	2	0	1	1	1	1	2	10
	SDR plus bulk fill composite (B)		0	1	3	1	0	0	1	4	10
	Silver Amalgam (C)		2	2	1	0	0	2	2	1	10
	Dycal (D)		4	0	0	0	2	2	0	0	8
Tissue disorganization (TO)	Glass ionomer cement (A)		2	2	0	1	1	1	1	2	10
	SDR plus bulk fill composite (B)		0	1	3	1	0	0	1	4	10
	Silver Amalgam (C)		2	3	0	0	0	2	2	1	10
	Dycal (D)		4	0	0	0	2	2	0	0	8
Reactionary dentin formation (RDF)	Glass ionomer cement (A)		5	0	0	0	5	0	0	0	10
	SDR plus bulk fill composite (B)		5	0	0	0	5	0	0	0	10
	Silver Amalgam (C)		5	0	0	0	5	0	0	0	10
	Dycal (D)		4	0	0	0	4	0	0	0	8

### 3.1 Inflammatory cell response and tissue disorganization

At a cavity depth of 2 mm, the Kruskal-Wallis test yielded a significant difference in Inflammatory Cell Reaction (ICR) and Tissue disorganization (TO) scores among the materials with a p-value of 0.028 and 0.017, respectively (Table 3). The post-hoc Dunn's test using a Bonferroni corrected alpha of 0.008 indicated that the mean rank ICR and TO score of only SDR plus composite and control group significantly differed with a p-value of 0.003 and 0.002, respectively (Table 4).

**Table 3:** Intergroup and Intragroup comparison of histological variables.

Histological variable	Inflammatory cell reaction							Tissue disorganization						
Material	2.5 mm cavity depth			2 mm cavity depth			Wilcoxon test	2.5 mm cavity depth			2 mm cavity depth			Wilcoxon test
	Mean	Median	Mean rank score	Mean	Median	Mean rank score		Mean	Median	Mean rank score	Mean	Median	Mean rank score	
Subgroup A1: GIC	1.80	2.00	10.1	1.00	1.00	9.9	z = 1.41, p = 0.157	1.80	2.00	10.1	1.00	1.00	10.1	z = 1.41, p = 0.157
Subgroup B1: SDR PLUS composite	2.80	3.00	14.9	2.00	2.00	15.2	z = 1.63, p = 0.102	2.80	3.00	14.9	2.00	2.00	15.6	z = 1.63, p = 0.102
Subgroup C1: AMALGAM	1.80	2.00	9.8	0.80	1.00	9.3	z = 1.63, p = 0.102	1.80	2.00	9.8	0.60	1.00	8.7	z = 1.89, **p = 0.059**
Subgroup D1: CONTROL	0.50	0.50	4	0.00	0.00	4.5	z = 1.41, p = 0.157	0.50	0.50	4	0.00	0.00	4.5	z = 1.41, p = 0.157
Kruskal Wallis test	chi sq = 9.06, **p = 0.028**			chi sq = 9.09, **p = 0.028**				chi sq = 9.06, **p = 0.028**			chi sq = 10.14, **p = 0.017**

**Table 4:** Intergroup Post Hoc paired comparisons of histological variables.

Variables	Inflammatory Cell Reaction				Tissue Disorganization			
Group Pair	2.5 mm cavity		2 mm cavity		2.5 mm cavity		2 mm cavity	
	Mean Rank difference	p-value	Mean Rank difference	p-value	Mean Rank difference	p-value	Mean Rank difference	p-value
SDR Plus vs GIC	4.8	0.2	5.3	0.1	4.8	0.2	5.5	0.1
GIC vs Amalgam	0.3	0.9	0.6	0.9	0.3	0.9	1.4	0.7
GIC vs Control	6.1	0.09	5.4	0.1	6.1	0.09	5.6	0.1
SDR PLUS vs Amalgam	5.1	0.1	5.9	0.08	5.1	0.1	6.9	0.04
SDR PLUS vs Control	10.9	**0.003**	10.7	**0.003**	10.9	**0.003**	11.1	**0.002**
AMALGAM vs Control	5.8	0.1	4.8	0.2	5.8	0.1	4.2	0.2

At a cavity depth of 2.5 mm, a Kruskal-Wallis test showed a significant difference in ICR and TO scores among the materials. The Post-Hoc Dunn's test indicated that only SDR plus composite and the control group significantly differed, with a p-value of 0.003(Table 4).

Application of the Wilcoxon test on mean ICR and TO score for intragroup comparison revealed that none of the materials tested indicated statistically significant differences between the ICR scores at two cavity depths. However, the marginal p-value (z = 1.89, p = 0.059) for Amalgam in TO score suggests further investigation or consideration of additional factors.

### 3.2 Reactionary dentin formation

No reactionary dentin formation was seen in any specimen at both cavity depths.

## 4 Discussion

Restorative dentistry focuses on treating tooth defects to control disease and restore function and aesthetics without compromising biological integrity. Remaining Dentin Thickness (RDT) is crucial for protecting the dental pulp during tooth preparation techniques and using restorative materials. Restorative procedures can result in pulp damage from various causes, including bacterial presence, toxicity, and thermal injury. Many restorative materials, such as resin cement, amalgam, zinc polycarboxylates, and zinc phosphates, can cause an inflammatory pulp response due to ingression through open dentinal tubules during cavity preparation. Minimizing pulp injury during cavity preparation and restoration is an important clinical goal, and clinicians must assess the potential harmful individual effects of cavity preparation, conditioning, and restoration.

The study included healthy premolar teeth planned for orthodontic extraction in individuals aged 15–18, which ensured a uniform population with teeth exhibiting similar histological characteristics and healing potential. All the procedures were done under rubber dam isolation to eliminate the risk of bacterial infection from the oral fluids.

In the present study, Class V cavities were prepared to evaluate the biocompatibility of materials, similar to a study done by *Dejou* et al.(1993)^19^ The Class V cavity was selected because of its proximity to the pulp chamber and as the buccal odontoblast layer (BOL) is thicker than the lingual odontoblast layer (LOL). Hence, BOL is a better choice for histologically studying the influence of restorative material on the odontoblastic layer.

The cavity dimensions were standardized to 2mm width and 2 mm height to homogenize the surface area parameter among the present study groups. *Trivedi* et al.*(2022)*,^4^ *Lopes* et al.(2009)^20^ and *Love* et al.(2002)^21^ have shown in their studies that inflammatory cell reactions can also directly depend on the prepared cavity's surface area. Heat generation during cavity preparation can also result in pulp inflammation.^16^ However, the data in Table 2 suggests that the clinical cavity preparation procedure did not lead to pulp damage, which was evident from the samples of the Control Group (D), where no inflammation or tissue disorganization occurred in most of the samples at cavity depths of 2 mm and 2.5 mm. Single operator executed all the clinical procedures to avoid any interoperator bias. The operator did all cavity preparations with intermittent contact, under copious water spray, and the diamond bur used was replaced after every four-cavity preparation as worn-out burs might necessitate excessive pressure during tooth preparation, leading to undesired frictional heat generation that can potentially induce pulpal inflammation.^6^^,^^7^^,^^15^^,^^16^

Dental literature has frequently discussed values for remaining dentin thickness (RDT) as it is crucial for long-term clinical success and protection of the pulpal parenchyma. Various studies have employed radiographic or histomorphometric analysis to calculate the RDT values.^2^^,^^22^ *Berbari* et al. (2018*)* study showed an average of 20 % radiographic underestimation of the RDT compared with the measurements carried out on the macrophotographs of the histologic sections. Therefore, the present study used the more accurate histomorphometric method to measure remaining dentin thickness. Table 1 presents histomorphometric analysis of remaining dentin thickness (RDT) in experimental teeth with cavities prepared to standardized depth dimensions (2 mm or 2.5 mm). It shows no statistically significant difference in mean RDT at 2 mm and 2.5 mm cavity depth intergroup (p = 0.662 & p = 0.886, respectively). There was no substantial difference in the RDT values among different groups at the same cavity depth. The investigator observed from Table 1 that intragroup comparison of mean RDT between 2 mm and 2.5 mm depth showed a significant difference for all the groups (GIC: p < 0.001; SDR PLUS: p < 0.001; Amalgam: p < 0.001; Control: p = 0.001). The endodontic operator restored the same material in all cavities; the pulpal response observed varied intragroup due to significant differences in RDT.

The restoration of cavities with thin remaining dentin thickness (RDT) presents challenges to the material's biological behaviour and pulp tissue's response. Research indicates that having 0.5 mm of remaining dentin between the pulp and restorative material is adequate to protect the tissue from material-related aggressions. Cavities with less than 0.5 mm of RDT are classified as very deep and exhibit a higher inflammatory response and more tissue disorganization, with a reduction in surviving odontoblasts. According to Table 1, the mean RDT in this study at the cavity of depth 2.5 mm ranged from 346 μm to 433 μm, which qualified them under the deep cavity category. In Table 3, the intragroup comparison of GIC, Amalgam, SDR plus composite, and Dycal showed a higher mean score of histological variables at 2.5 mm cavity depth than 2 mm, which is in line with the studies that demonstrated mild pulp response and fewer reduction of odontoblast cells beneath an RDT >0.5 mm.^2^^,^^23^^,^^24^

In the present study, the histological inflammatory response to GIC, amalgam, SDR plus bulk fill composite, and Dycal was recorded as score 0 (no inflammation), score 1 (mild inflammation), score 2 (moderate inflammation), and score 3 (severe inflammation).^6^, ^7^, ^8^, ^9^, ^10^, ^11^

**Fig. 4:**
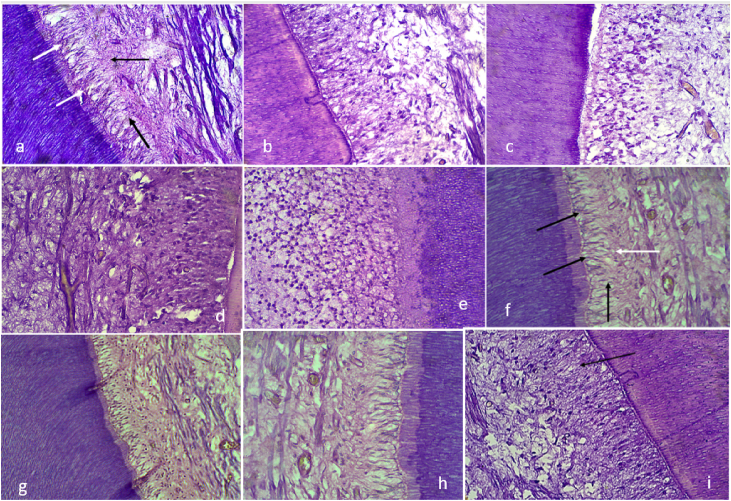
Histopathological examination of tooth sections. **Group A GIC restorations**The odontoblast layer related to the cavity floor was preserved (white oblique arrows). Note the normal cell-free (black vertical arrow) and cell-rich (black horizontal arrow) zones with no inflammatory pulp reaction (RDT = 1200 μm), b) Disruption of the odontoblast layer as well as slight pulp inflammation associated with discrete tissue disorganization was observed. Grade 1 inflammatory reaction (RDT = 801 μm), c**)** the odontoblast layer related to the cavity floor was slightly disrupted. GRADE 2 inflammatory reaction (RDT = 266 μm). **Group B SDR plus bulk fill restorations** d) The pulp layers were disorganized, and the subjacent pulp exhibits dilated and congested blood vessels (white arrow) among inflammatory mononuclear cells. (RDT = 895 μm), e) The pulp layers were completely disorganized, and the subjacent pulp exhibits a large number of inflammatory mononuclear cells (score 3 inflammatory response) (RDT = 301 μm). **Group C Amalgam restorations** f) The odontoblast layer related to the cavity floor was preserved (black oblique arrows). Note the normal cell-free (black vertical arrow) and cell-rich (white horizontal arrow) zones with no inflammatory pulp reaction (RDT = 1023 μm), g) Disruption of the odontoblast layer as well as slight pulp inflammation associated with mild tissue disorganization was observed. Score 1 inflammatory reaction (RDT = 564 μm). **Group D Control**, h) in Dycal lined tooth the pulp tissue subjacent to the cavity floor exhibited partial disruption of the odontoblast layer (arrows). Note the discrete disorganization of the adjacent pulp tissue in which mononuclear inflammatory cells (RDT = 289 μm), i) Intact sound premolar exhibited normal histological characteristics. Note the continuous and homogeneous odontoblast layer as well as the defined cell-free and cell-rich zones. H/E, × 400.

Concerning Table 2, the GIC group inflammatory response at 2 mm cavity depth shows that two specimens exhibited no pulp inflammation and a continuous and homogenous odontoblast layer with well-defined cell-free and cell-rich zones (Fig. 4a), two specimens presented mild inflammatory reactions associated with few polymorphonuclear cell infiltration (Fig. 4b) and one specimen observed a severe inflammatory response mediated by mononuclear cells and several dilated and congested blood vessels. At 2.5 mm cavity depth, one specimen exhibited no pulp inflammation, which can be due to the thicker remaining dentin (RDT = 688 μm) present compared to other specimens of the same subgroup. One specimen presented a mild inflammatory response associated with few polymorphonuclear cell infiltration. Two specimens displayed moderate inflammatory response mediated by a more significant number of mononuclear cells, and several dilated and congested blood vessels were observed with the absence of defined cell-free and cell-rich zones (Fig. 4c). Two specimens showed a severe inflammatory reaction, which can also be attributed to thin remaining dentin thickness (RDT = 132 μm and RDT = 266 μm). As evident from Table 2, most specimens showed none to a mild inflammatory reaction. There was no significant difference in the inflammatory cell reaction and tissue disorganization of GIC compared to the control group (Table 3). Hence, it can be implied that GC GIC has good biocompatibility. *Hii* et al.(2019),^25^ *Rodriguez* et al.(2013),^26^ and *Ribeiro* et al.(2020)^8^ made similar observations concerning biocompatibility.

The histological examination showed that Group B exhibited mild to severe inflammatory response. In this group, three samples exhibited moderate inflammatory response characterized by the increased number of mononuclear cells and complete disruption of the odontoblastic layer without pulp necrosis (Fig. 4d), followed by a severe response in one sample but a mild response in another sample with fewer polymorphonuclear cell infiltrations at a cavity depth of 2 mm. The mild response can be due to more than 1 mm of remaining dentine (RDT = 1046 μm). At 2.5 mm cavity depth, out of five samples, four samples showed severe inflammatory responses characterized by marked infiltration of mononuclear cells and complete disruption of the odontoblastic layer with pulpal necrosis (Fig. 4e). At the same time, one had moderate inflammatory responses with more mononuclear cells and dilated, congested blood vessels present on histological examination. Table 2 indicates that a maximum number of samples exhibited severe inflammatory reactions. The inflammatory cell reaction of the SDR plus bulk fill composite group showed a statistically significant difference from the control group at both 2 mm and 2.5 mm depths (p-value 0.003)(Table 2). This data implies that SDR plus bulk fill composite has poor biocompatibility to the dental pulp, which can be attributed to the cytotoxic effects of monomer Triethyleneglycoldimethacrylate (TEGDMA)^27^ and photoinitiator Camphorquinone,^28^ proven from previous studies.

In the present study, despite the minimum dentin thickness between the cavity floor and the pulp, the light-curing procedure using a 470 mW/cm2 light intensity for 40 s did not have a detrimental effect on the pulp as reported in some previous studies.^29^^,^^30^ The intact pulp tissue homeostasis in sound teeth may have prevented an increase in pulp temperature, attributing to this. However, it's important to note that teeth with pulp inflammation due to a carious lesion have their microcirculation downregulated and, hence, cannot reduce generated heat.^31^ Thus, while heat may be generated during the light irradiation of bulk fill composite, the adverse impact on pulp tissues is considered minimal.

According to Table 2, the samples in Group C (Amalgam) show that at 2 mm cavity depth, two specimens exhibited no inflammatory response (Fig. 4f), and two presented mild inflammatory reactions associated with few polymorphonuclear cell infiltration. However, one specimen displayed a moderate inflammatory response mediated by a more significant number of mononuclear cells, possibly due to the thin remaining dentin present (RDT = 695 μm) compared to other samples of the same subgroup. At 2.5 mm cavity depth, out of five samples, nearly all showed mild (2 samples) to moderate (2 samples) inflammatory response, evident from the histological examination. The histological slide, which depicted a slight increased number of polymorphonuclear cell infiltration, was included in a mild inflammatory reaction (Fig. 4g). In contrast, the one with a more significant number of mononuclear cells and several dilated and congested blood vessels was categorized under moderate inflammatory response, however, the thin remaining dentin (RDT = 215 μm) and the heat-conducting property of amalgam combined to attribute a severe inflammatory reaction in one of the specimens. Most of the specimens showed mild to moderate inflammatory responses. As Table 3 shows, there was statistically no significant difference in the inflammatory cell reaction of amalgam compared to the control group (p-value = 0.1). Therefore, one can infer that amalgam demonstrates good biocompatibility.

In support of our study, *Chandwani* et al.(2014)^9^ made similar observations, while Manley(1941)^32^ observed that silver amalgam produced a definite reaction in pulp under large and deep cavities. For this reason, a cavity lining of a non-irritating and non-conducting material is always advisable to ensure proper insulation. Araujo et al.^12^ found that mercury released from dental amalgam restorations does not contribute to systemic disease or toxicological effects.

In the control group with Dycal lining, data from Table 2 displays that all the specimens evaluated at 2 mm cavity depth presented no inflammation or tissue disorganization. In comparison, at 2.5 mm cavity depth, two specimens showed no inflammation or tissue disorganization, while the other two specimens exhibited mild inflammatory pulp response and tissue disorganization (Fig. 4h). Thin dentin remaining under the cavity (RDT = 289 μm and 312 μm) might have caused the inflammation. As evident from Table 2, most specimens displayed no inflammatory pulpal response.

The current study evaluated the survival of odontoblasts under tissue disorganization criteria as it helps in tertiary dentin formation.^33^^,^^34^ The composite was observed to have the maximum detrimental effect on the odontoblastic layer compared to GIC and Amalgam (Table 4). A significant difference in disruption of the odontoblastic layer was seen with SDR plus bulk fill composite compared to the control group at 2 mm (p value = 0.002) and 2.5 mm (p value = 0.003) cavity depth. In cases with no or mild disorganization of tissue, surviving odontoblast would lay down reactionary dentin, which has better quality than reparative dentin.^33^

In the present study, reactionary dentin formation was included as a criterion. According to an animal model study, most of the damaged odontoblasts degenerated one day after cavity preparation, and reactionary dentin deposition occurred five days after cavity preparation.^35^ However, in the present study, no reactionary dentin formation was seen at the time interval of 7 days in all the groups (Table 2). Many histological studies evaluating pulp response under various restorative materials also demonstrated the influence of postoperative time, as pulpal response often decreases over time.^9^

The results suggest that remaining dentin thickness below 0.5 mm can result in a moderate to severe inflammatory reaction with a contraction in the odontoblastic layer in the pulp. SDR plus bulk fill composite showed significantly higher inflammatory reaction and disruption of the odontoblastic layer than the control group. Amalgam remains the more mechanically and biologically competent material for restoring teeth, particularly in cases where esthetics are not the primary concern. Conventional glass ionomer cement is biologically compatible with esthetic restoration but is limited to non-stress-bearing areas.

The study has limitations, such as a relatively small sample size and the use of sound teeth without pre-existing inflammation, which may not fully replicate clinical scenarios. Further randomized clinical trials are needed to determine the role of SDR plus bulk fill composite in pulp regeneration and provide more insight into the clinical effectiveness of these materials in real-world scenarios.

## 5 Conclusion

The study concluded that critical RDT varies for different restorative materials. It was observed that both glass ionomer cement and amalgam demonstrated acceptable biocompatibility when used in deep cavities. At the same time, SDR plus bulk fill composite proved to be the least biocompatible. This study reaffirmed that amalgam remains the more mechanically and biologically competent material for restoring teeth, particularly in cases where esthetics are not the primary concern.

## Funding

This research did not receive any specific grant from funding agencies in the public, commercial, or not-for-profit sectors.

## Declaration of competing interest

The authors declare that they have no known competing financial interests or personal relationships that could have appeared to influence the work reported in this paper.
